# Organic Cation Engineering for Vertical Charge Transport in Lead‐Free Perovskite Quantum Wells

**DOI:** 10.1002/smsc.202000024

**Published:** 2021-02-07

**Authors:** Ke Ma, Sheng-Ning Hsu, Yao Gao, Zitang Wei, Linrui Jin, Blake P. Finkenauer, Libai Huang, Bryan W. Boudouris, Jianguo Mei, Letian Dou

**Affiliations:** ^1^ Davidson School of Chemical Engineering Purdue University West Lafayette IN 47907 USA; ^2^ Department of Chemistry Purdue University West Lafayette IN 47907 USA; ^3^ Birck Nanotechnology Center Purdue University West Lafayette IN 47907 USA

**Keywords:** 2D tin perovskites, charge transfer between quantum wells, conjugated thiophene ligands, lead-free perovskite quantum wells

## Abstract

2D organic–inorganic hybrid halide perovskites are promising semiconductor materials for a variety of device applications. However, fundamental issues of charge transport through multiple quantum wells separated by bulky organic ligands remain unsolved. Herein, a mixture of π‐conjugated organic ligands, (2‐(3‴,4′‐dimethyl‐[2,2′:5′,2′:5″,2‴‐quaterthiophen]‐5‐yl)ethan‐1‐ammonium iodide) (4Tm) and 2‐([2,2′‐bithiophen]‐5‐yl)ethan‐1‐aminium iodide (2T), is used as spacer to form (4Tm)_*x*_(2T)_2−*x*_SnI_4_ 2D perovskite thin films. The new strategy of alloying 2T into 4Tm ligands reduces the interlayer distance (barrier thickness) while maintaining the relatively small energy barrier height for the hybrid quantum wells, enhances interlayer interactions, and improves charge transport across adjacent perovskite layers. Moreover, solar cell devices fabricated with (4Tm)_*x*_(2T)_2−*x*_SnI_4_ exhibit improved photovoltaic properties and stability.

## Graphical abstract

2D organic–inorganic hybrid halide perovskites improve the stability of perovskite photovoltaic devices, but suffer from inefficient charge transfer through multiple quantum wells. Alloyed π‐conjugated organic ligands are used as spacers to shorten the interlayer distances and lower energy barrier heights simultaneously to achieve promising optoelectronic properties. The conjugated ligands also improve the stability of the fabricated devices.

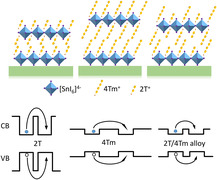

## 1 Introduction

Perovskite solar cells (PSCs) have garnered considerable attention in the past ten years, reaching a record power conversion efficiency (PCE) of over 25% with an astonishing speed.^[^ ^1^ ^]^ However, the large‐scale fabrication of 3D lead‐based PSCs is restricted by the toxicity of lead and the intrinsic instability of the materials.^[^ ^2^, ^3^, ^4^ ^]^ Thus, more recent efforts have focused on solving these bottleneck issues for commercialization.^[^ ^5^, ^6^ ^]^ Alternative light‐absorbing materials have been extensively explored, such as tin (Sn)‐, antimony (Sb)‐, and bismuth (Bi)‐based perovskites.^[^ ^4^, ^7^, ^8^, ^9^ ^]^ Among these, the narrower bandgap and higher carrier mobility of Sn‐based perovskites make them promising for high‐performance PSCs.^[^ ^10^ ^]^ However, one major drawback of Sn PSCs is their low stability toward oxygen and moisture. The instantaneous oxidation of Sn^2+^ to Sn^4+^ leads to self‐doping of this material and a transforming of the electronic properties such that they are closer to those of metals instead of semiconductors; in turn, this leads to time‐dependent (and, ultimately, poor) device performance. This behavior not only induces a long‐term stability issue for Sn PSCs, but also complicates the fabrication process for achieving high‐quality films.^[^ ^11^ ^]^ Using 2D perovskites is an effective way of obtaining high device stability.^[^ ^12^, ^13^, ^14^, ^15^, ^16^, ^17^, ^18^ ^]^ Partially or completely replacing small organic cations (e.g., methylammonium (MA) or formamidinium (FA)) with large ammonium cations (e.g., butylammonium (BA) or phenylethylammonium (PEA)) confines the metal halide octahedra into single‐ or multi‐perovskite slabs and protects them against moisture and oxygen.^[^ ^15^ ^]^

For most reported 2D Sn perovskites, the selection of the large organic ligand is limited to simple alkyl or phenyl derivatives. These ligands have wide bandgaps that induce large potential barriers in the perovskite. As a result, the out‐of‐plane charge transport is inefficient.^[^ ^19^ ^]^ To address these intrinsic stability and charge transport issues, we introduce π‐conjugated oligothiophene ligands into *n* = 1 L_2_SnI_4_ (L represents large ammonium cations) perovskites and investigate their impact on the performance of 2D PSCs. It is known that conjugated organic semiconductors possess higher dielectric constants comparing to conventional organic ligands, and thus have improved carrier mobility in 2D perovskites.^[^ ^20^, ^21^, ^22^ ^]^ However, they also possess bulky size and introduce large interlayer distance and this large distance decreases the carrier tunneling probability. To address this issue, we further designed an alloy system by mixing a bulky small‐bandgap ligand 2‐(3‴,4′‐dimethyl‐[2,2′:5′,2′:5″,2‴‐quaterthiophen]‐5‐yl)ethan‐1‐ammonium iodide (4Tm) and a less bulky wide‐bandgap ligand 2‐([2,2′‐bithiophen]‐5‐yl)ethan‐1‐aminium iodide (2T) as the spacers (chemical structures are shown in Figure S1, Supporting Information). In this way the interlayer distance and molecular interaction can be further tailored. The alloyed ligand system with composition of (4Tm)(2T)SnI_4_ exhibits more efficient out‐of‐plane carrier transport compared to pure ligand systems due to the higher carrier mobility, weaker space charge effect, and longer carrier lifetime. This effect can boost the photocurrent density and results in improved photovoltaic (PV) properties. The fabricated PSCs show extraordinary thermal and moisture stability compared to their 3D counterparts. Our results provide new fundamental investigations on the charge transport mechanism in 2D halide perovskite quantum wells and provide practical insights into future improvement strategies.

## 2 Results and Discussion

### 2.1 Structural and Optical Properties

**Figure 1:**
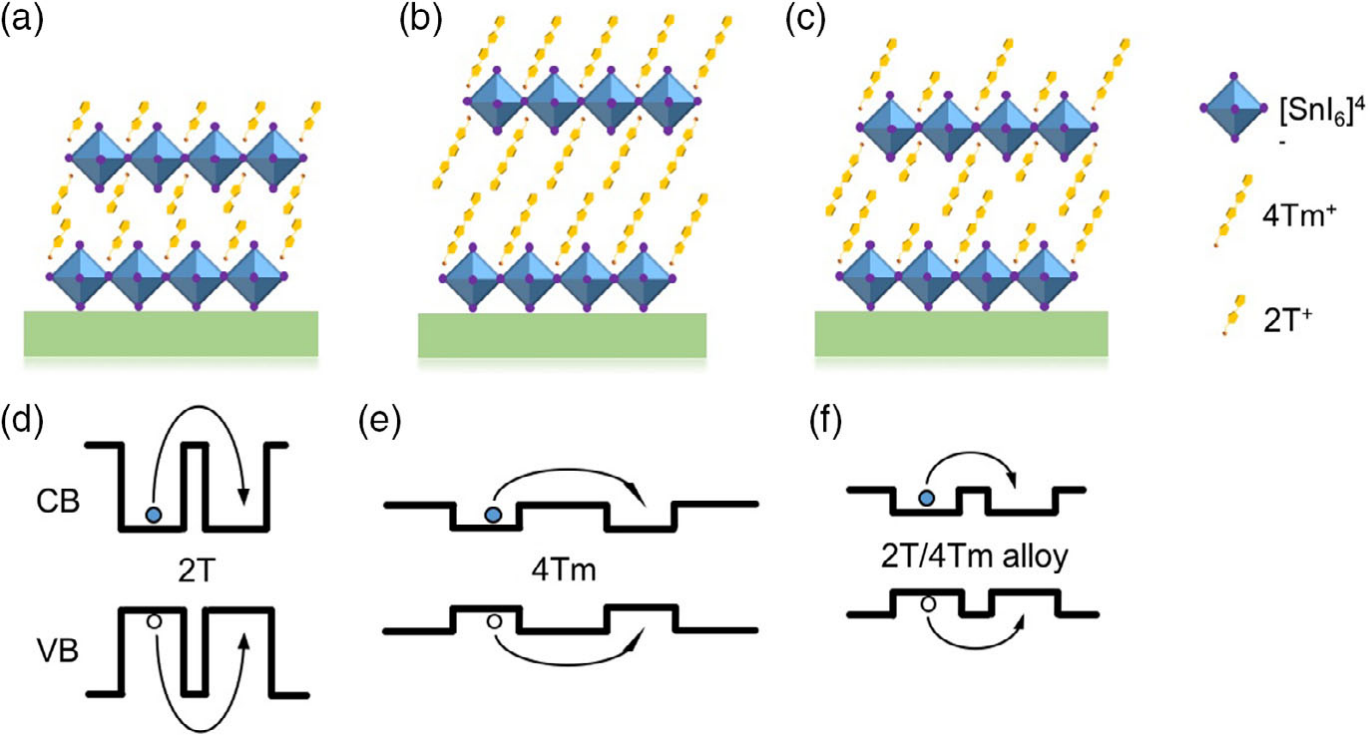
2D perovskite film structure scheme: Schematic crystal structure of the layered 2D a) (2T)_2_SnI_4_, b) (4Tm)_2_SnI_4_, and c) (4Tm)(2T)SnI_4_ perovskite thin films. Schematic illustration of the charge transfer process in multicomponent quantum wells: d) (2T)_2_SnI_4_, e) (4Tm)_2_SnI_4_, and f) (4Tm)(2T)SnI_4_ 2D perovskite, for ligands with different lengths and bandgaps.

2D perovskite quantum well thin films with compositions of (4Tm)_*x*_(2T)_2−*x*_SnI_4_ were fabricated using oft‐used spin‐coating methods. As schematically shown in **Figure** **1**a–c, the structure can be viewed as stacked 2D Sn‐perovskite slabs sandwiched by organic ligand layers. The organic ligand layers introduce barriers for out‐of‐plane charge transfer between perovskite quantum wells. As shown in Figure 1d–f, the barrier distance and barrier height, which are determined by the organic ligand layers, are two key factors of this charge tunneling process.^[^ ^23^ ^]^ Here, 2T and 4Tm are selected as two ligands to be studied because of their similar oligothiophene moieties but different electronic and structure properties.^[^ ^24^ ^]^ 2T has a smaller size but a larger bandgap, which can cause a higher energy barrier between quantum wells (Figure 1d), whereas 4Tm has a smaller bandgap but a longer oligothiophene tail, which will introduce a larger interlayer space and thus larger barrier distance (Figure 1e). The designed alloyed‐ligand systems are expected to combine the advantages of each pure‐ligand system to lower both barrier height and barrier distance, and achieve a more efficient charge transfer process between quantum wells (Figure 1f).

**Figure 2:**
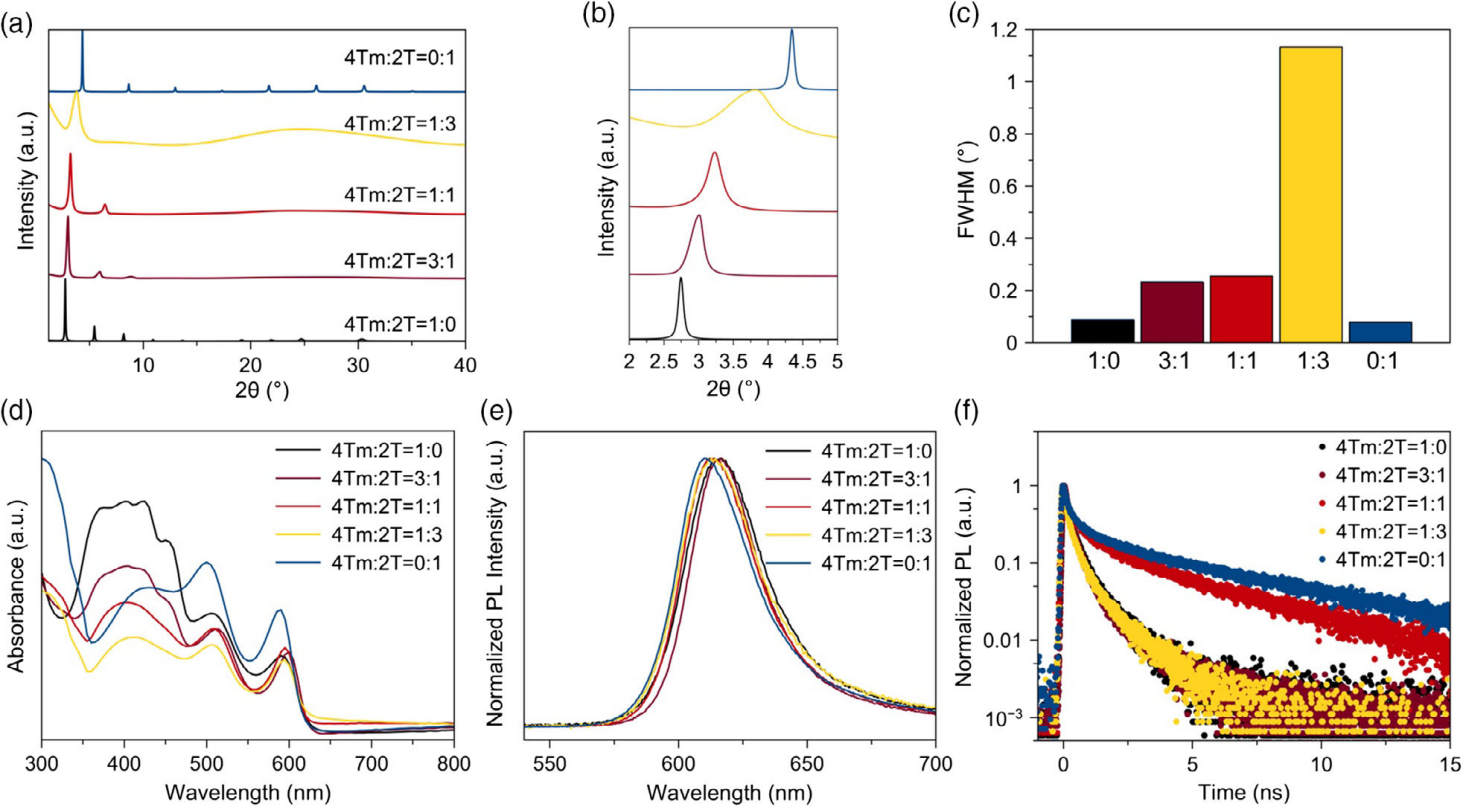
2D perovskite films structure, morphology, and optical property characterization: a) XRD spectra, b) magnified XRD patterns at low angles, and c) FWHM calculated from (001) crystal planes of 2D perovskites with different 4Tm:2T ratios. d) UV–vis absorption spectra, e) steady‐state PL spectra, and f) time‐resolved PL spectra of 2D perovskite films on glass substrates.

2D perovskites with *n* = 1 composition are known to adopt an orientation parallel to the substrates, which is confirmed in our case using X‐ray diffraction (XRD) (**Figure** **2**a).^[^ ^25^ ^]^ Diffractograms of the perovskite films on glass/ITO/PEDOT:PSS (ITO: indium tin oxide; PEDOT:PSS: poly(3,4‐ethylenedioxythiophene):poly(styrenesulfonate)) substrates revealed low‐angle lattice reflections corresponding to the (001) crystal planes. The diffraction angles shifted to higher angles with the 4Tm:2T ratios in the alloyed‐ligand system decreasing. The calculated interlayer distances between the perovskite slabs changed from 32.2 to 30.1, 27.4, 23.1, and 20.3 Å as the 4Tm:2T ratio changed from 1:0 to 3:1, 1:1, 1:3, and 0:1 (denoted as 4Tm, 3:1, 1:1, 1:3, and 2T samples). The full width at half maximum (FWHM) increased when the 2T and 4Tm were mixed in the alloyed‐ligand systems, indicating the increased disorder of interlayer distances in the alloyed‐ligand systems compared to pure‐ligand systems (Figure 2b,c). However, no diffraction peaks that are associated with (4Tm)_2_SnI_4_ or (2T)_2_SnI_4_ were observed in the alloyed‐ligand films, indicating the dominant phase likely adopts an interdigitated molecular packing with mixed 4Tm and 2T ligands in the organic layers, as shown in Figure 1c, without phase obvious segregation. Among the three alloyed‐ligand samples, the 3:1 and 1:1 samples have a relatively narrow FWHM, which explains the preferable crystal structure.

The optical UV–vis absorbance data show typical 2D perovskite spectra with a high‐energy continuum absorption edge and a low‐energy excitonic peak (Figure 2d). The strong absorption from the 350 to 480 nm region in (4Tm)_*x*_(2T)_2−*x*_SnI_4_ arises from the absorption of 4Tm ligands. All (4Tm)_*x*_(2T)_2−*x*_SnI_4_ samples exhibit similar excitonic peaks and only slight redshifts are observed in the alloyed‐ligand samples. Incorporation of 2T into the (4Tm)_2_SnI_4_ lattices causes a slight blueshift in photoluminescence (PL) peaks (Figure 2e). The exciton binding energies of (4Tm)_*x*_(2T)_2−*x*_SnI_4_ extracted from the Tauc plot (Figure S2, Supporting Information) are within the range 140–190 meV.^[^ ^26^ ^]^ We noted that these exciton binding energies are small with respect to other 2D perovskites in the literature,^[^ ^5^, ^19^, ^27^ ^]^ which is likely due to weaker dielectric confinements of the π‐conjugated organic ligands compared to other ligands. The 1:1 sample exhibits the smallest exciton binding energy (178 meV) among the alloyed‐ligand samples, which creates a precondition for stronger exciton–exciton coupling and weaker quantum confinement.

To characterize the carrier dynamics in these thin films, we used time‐resolved PL (TRPL) spectroscopy measurements with the samples coated on glass substrates (Figure 2f). All the species have relatively short carrier lifetimes compared with other perovskite materials, which is typical for *n* = 1 2D perovskites.^[^ ^25^ ^]^ Among all species, the 1:1 and (2T)_2_SnI_4_ samples exhibit obvious longer PL decay time. We speculate that the long PL decay time in the (2T)_2_SnI_4_ sample originates from the high crystallinity of the film prepared under the same annealing conditions as (4Tm)_2_SnI_4_. The long PL decay time in the 1:1 sample is ascribed to the efficient electron and hole separation, relatively low trap density compared to other alloyed‐ligand samples, and improved carrier mobility, which will be discussed in detail subsequently.

For all the (4Tm)_*x*_(2T)_2−*x*_SnI_4_ films, smooth and pinhole‐free perovskite films are achieved with 100 °C thermal annealing after spin coating (Figure S3, Supporting Information). While higher annealing temperatures can increase the crystallinity and grain size, the large pinhole density and potential oxidation of Sn^2+^ species impede the application in PSCs (Figure S4, Supporting Information). Although determination of the grain size is difficult, these data indicate no obvious difference in surface morphology among these materials, with small grains below 100 nm.

### 2.2 PV Properties

**Figure 3:**
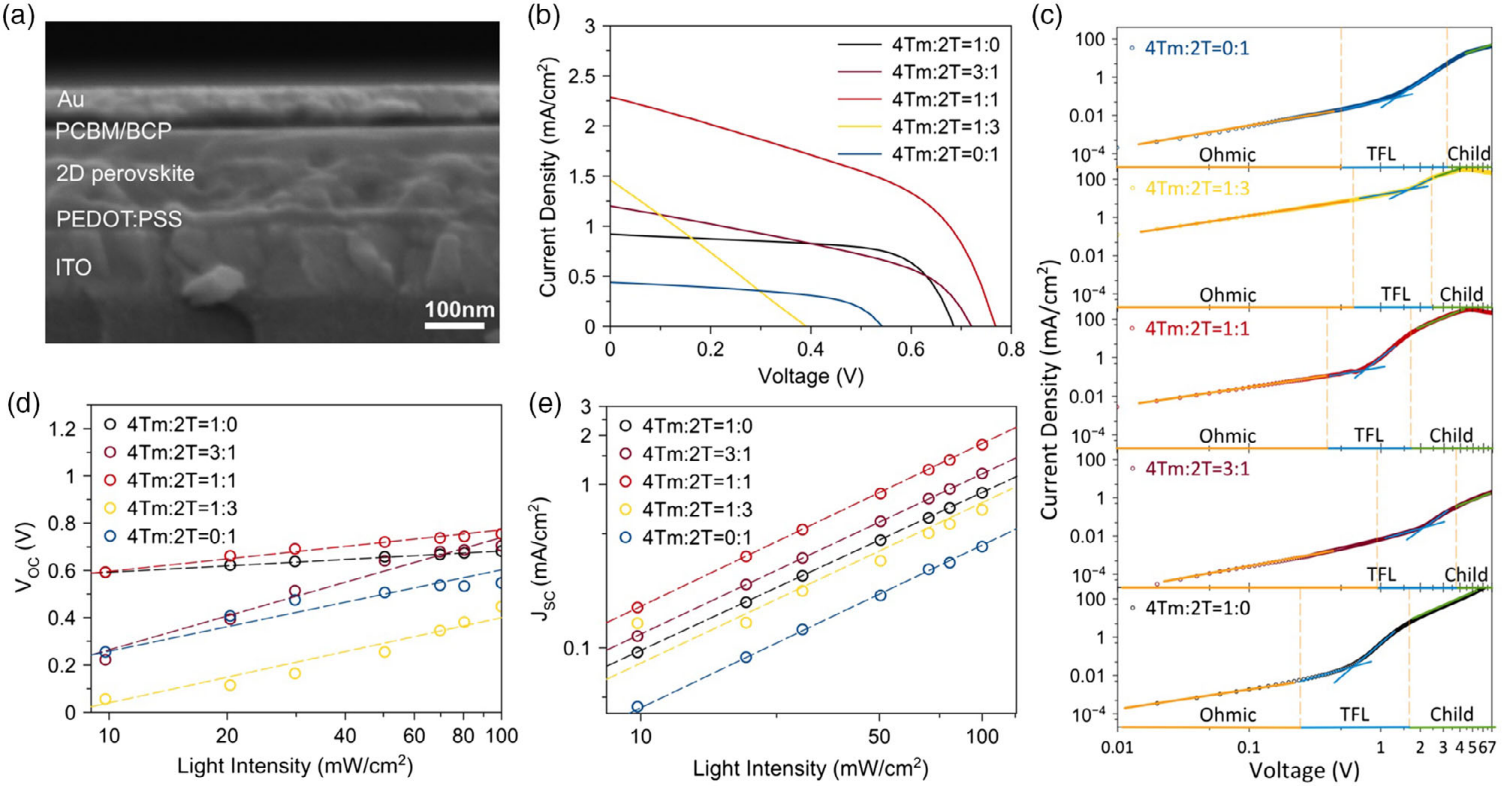
2D perovskite PV properties and charge transfer dynamics. a) Cross‐sectional SEM image of (4Tm)(2T)SnI_4_ 2D PSC. b) Current density–voltage (*J–V*) curves under an AM 1.5G solar simulator for champion devices based on 2D perovskite. c) Current density–voltage curves of hole‐only devices for 2D perovskites. The hole‐only device structure is ITO/PEDOT:PSS/PVSK/PTAA/Au. d) *V*_OC_ versus light intensity in a seminatural logarithmic scale curve of 2D PSCs. e) *J*_SC_ versus light intensity on a double‐logarithmic scale curve of 2D PSCs.

To compare how the alloyed‐ligand systems in 2D perovskites affect the PV properties, we fabricated planar PSCs with these thin films. We adopted an inverted planar solar cell structure with a configuration of ITO/PEDOT:PSS/2D perovskite/PCBM/BCP/Au (PCBM: [6,6]‐phenyl‐C61‐butyric acid methyl ester; BCP: bathocuproine), as shown in the cross‐sectional SEM image in **Figure** **3**a. The current density–voltage (*J–V*) curves of (4Tm)_*x*_(2T)_2−*x*_SnI_4_ PSCs were recorded under an AM1.5G solar simulator (Figure 3b). The (4Tm)_2_SnI_4_ PSC yields a PCE of 0.41%, with *V*_OC_ of 0.68 V, short‐circuit current density (*J*_SC_) of 0.92 mA cm^−2^, and FF of 68.48%. The (2T)_2_SnI_4_ device shows significantly lower performance with a PCE of 0.12% due to the larger bandgap and higher energy barrier height of 2T ligands. Interestingly, all the alloyed‐ligand devices exhibit improved *J*_SC_, and devices with 3:1 and 1:1 samples also show improved *V*_OC_. The best device was achieved with the 1:1 sample, with a PCE of 0.80%, *V*_OC_ of 0.77 V, *J*_SC_ of 2.29 mA cm^−2^ and FF of 45.80%. The device shows small hysteresis (Figure S5, Supporting Information), which is largely associated with the suppression of ion migration by using these bulky conjugated ligands.^[^ ^28^ ^]^ The nonobvious photodiode property of the device with the 1:3 sample is most likely due to the low crystallinity of the film, which has been indicated in the XRD spectra (Figure 2a–c). Although both the (4Tm)_2_SnI_4_ and (2T)_2_SnI_4_ devices exhibit high *V*_OC_ and high FF among current 2D Sn PSCs, the major limitation factor here is the *J*_SC_.^[^ ^14^, ^29^, ^30^, ^31^, ^32^ ^]^ The enhancement of *J*_SC_ in the alloyed‐ligand PSCs contributes significantly to the improved PCE (vide infra).

### 2.3 Defect Behavior and Charge Transfer Dynamics

To further investigate the effect of the alloyed‐ligand system on the PV properties of the 2D perovskite, we studied the defect behavior and charge transfer dynamics in the devices. The results of space‐charge‐limited‐current (SCLC) measurements on hole‐only devices (ITO/PEDOT:PSS/2D perovskite/PTAA/Au, PTAA: poly[bis(4‐phenyl)(2,4,6‐trimethylphenyl)amine) are shown in Figure 3c. We used the trap‐filled limit voltage (*V*_TFL_) in SCLC measurements to evaluate the trap density of each film. As shown in Figure 3c, the lowest trap density (or *V*_TFL_) is achieved from the (4Tm)_2_SnI_4_ device, with a value of 5.882 × 10^16^ cm^−3^ (0.61 V). Among the three alloyed‐ligand samples, the lowest trap density 7.206 × 10^16^ cm^−3^ (0.75 V) is obtained with the 1:1 sample. To further validate the charge recombination behavior, light‐intensity‐dependent *J–V* measurements were acquired. As described in Figure 3d, the slopes in *V*_OC_ versus light intensity curves show 1.33*k*_B_ *T/q* for the (4Tm)_2_SnI_4_ and 2.36*k*_B_ *T/q* for the 1:1 sample as two of the lowest values. These results demonstrate that the (4Tm)_2_SnI_4_ and 1:1 samples have better capability to suppress trap‐assisted recombination in the devices, which is in agreement with the observation in SCLC measurements. However, the 1:1 sample still contains higher trap density in the devices compared to (4Tm)_2_SnI_4_. The low trap density of the 1:1 sample among all the alloyed‐ligand species might be due to its well‐matched interdigitating ligand packing.

In addition to trap density characterization, hole mobility can also be extracted from the SCLC measurements. By fitting the curve by the Mott–Gurney Law in Child's regime (*J∝V* ^2^) at high voltage, the hole mobility is collected as 1.263 × 10^−5^ cm^2^·s^−1^·V^−1^ for the 1:1 device, which is nearly two times that of (4Tm)_2_SnI_4_ (0.666 × 10^−5^ cm^2^·s^−1^ V^−1^) and astonishingly 20 times that of (2T)_2_SnI_4_ (0.058 × 10^−5^ cm^2^·s^−1^ V^−1^). In addition to decreasing the interlayer distance, the insertion of 2T into 4Tm packing also weakens the steric hindrance and restriction of the perovskite lattice, and provides more space and flexibility for the 4Tm ligand in the perovskite structure, as shown in Figure 1c. This allows stronger interaction between 4Tm ligands of two adjacent organic layers and likely enhances the π‐orbital overlapping in the out‐of‐plane direction, and thus could further improve hole mobility.^[^ ^28^ ^]^ Although the 1:3 device also shows high hole mobility (1.217 × 10^−5^ cm^2^·s^−1^ V^−1^), the large defect density in the film and poor film quality inhibit it from obtaining an overall good PV property. We also found a power‐law dependence of *J*_SC_ with light intensity (*J∝φ* ^*α*^) in the double‐logarithmic scale curve (Figure 3e), giving *α* values of 0.967, 0.972, and 0.988 for (4Tm)_2_SnI_4_, (2T)_2_SnI_4_, and 1:1 devices, respectively. The ideal solar cell has an *α* value of 1, indicating that charge‐collection efficiency is independent of light intensity. Here, all *α* values are close to 1, illustrating the dominant recombination process is monomolecular recombination; the α value in the 1:1 device is closer to 1, which indicates a weaker space charge effect due to reduced carrier accumulation.^[^ ^33^ ^]^ This facilitates effective carrier transport, and it is in agreement with the hole mobility results and the good PV properties in the 1:1 device. Therefore, among all the pure‐ligand and alloyed‐ligand systems, the 1:1 sample obtains the best overall PV performance due to its relatively low exciton binding energy and low trap density, longer PL decay time, and most importantly the effective charge transport process. Our ligand‐alloying approach is a simple and effective method for utilizing the semiconducting property of conjugated ligands to achieve better PSCs. Further improvements are expected via enhancing thin film crystallinity and better molecular design.

To gain a deeper insight into the charge transfer process in these 2D PSCs, we further investigated the influence of the active layer thickness on the PV performance. As shown in Figure S6, Supporting Information, devices using thinner films exhibit better PCEs with slightly enhanced *V*_OC_ and FF and greatly increased *J*_SC_. The type‐I quantum well alignment (Figure 1e) stacks throughout the film thickness, in which the charge transport between inorganic slabs depends on a tunneling process mediated by the organic layer. Therefore, dissociated excitons will go through a trapping–detrapping process, which is largely driven by the internal field, to reach the interface between the perovskite and charge collection layers (Figure S6, Supporting Information).^[^ ^34^ ^]^ Increasing the thickness of the films enlarges the distance between the electrodes and decreases the internal field across the films, neither of which is preferred for efficient charge transport. Although thicker absorbers can increase the number of photons absorbed by the devices, the charge transfer efficiency might peak; then, excess carriers generated in the devices cannot be transferred and extracted efficiently. Moreover, because the thin absorbers can be fully depleted by the internal field, traps in the bulk are less essential as the recombination mainly occurs at the interfaces. This explains why the slightly higher trap density in the 1:1 sample compared to that in (4Tm)_2_SnI_4_ does not induce a deleterious effect on PV performance.

### 2.4 Stability

**Figure 4:**
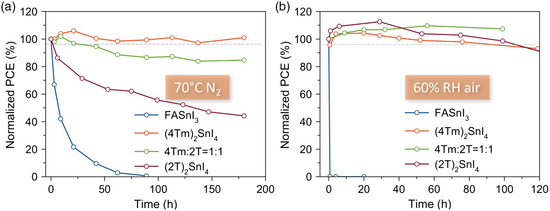
Stability of 2D PSCs. Normalized PCE variation curves of different PSCs without encapsulation, exposed to a) 70 °C in nitrogen and b) 60% relative humidity (RH).

To assess the stability of the PSCs, we fabricated (4Tm)_2_SnI_4_ and (4Tm)(2T)SnI_4_ quantum well PSCs, and used 3D FASnI_3_ devices for comparison. The *J–V* curve of the pristine FASnI_3_ device is shown in Figure S7, Supporting Information. All devices were tested without encapsulation. As shown in **Figure** **4**a, the FASnI_3_ device degrades exponentially during continuous heating at 70 °C in a N_2_ atmosphere, and it fails completely within 90 h. In contrast, the 1:1 device retains 85% of its initial PCE, and the (4Tm)_2_SnI_4_ device retains 100% of its initial PCE after 180 h heating. The thermal stability of the (2T)_2_SnI_4_ device decreased compared to that of the (4Tm)_2_SnI_4_ device, which indicates stronger interactions between the 4Tm and SnI_6_ octahedra lattice. Moreover, moisture stability tests were conducted in air with 60% relative humidity (Figure 4b). Whereas the FASnI_3_ device becomes completely dysfunctional within 1 h, all three devices fabricated with conjugated thiophene ligands exhibit high humidity stability. The (4Tm)_2_SnI_4_ and (2T)_2_SnI_4_ devices retain 93% and 91% of their initial PCE after 120 h, respectively. For the 1:1 device, the enhanced PCE, induced by an aging effect, did not drop back to its initial PCE after 100 h exposure under humidity. The remarkable stability of the 2D perovskite devices is largely due to the hydrophobicity of the conjugated thiophene ligands, the strong interactions between ligands and SnI_6_ octahedra, and the avoidance of volatile components.^[^ ^28^ ^]^ This hypothesis is also supported by X‐ray photoelectron spectroscopy (XPS) characterization. Storing the (4Tm)_2_SnI_4_ thin film in air caused only 6% of the initial Sn^2+^ to be oxidized (Figure S8, Supporting Information), whereas the FASnI_3_ film decomposed completely, and no XPS data were obtained. These extraordinary device stabilities induced by π‐conjugated organic spacers emphasize the importance of developing organic ligands for 2D PSCs.

## 3 Conclusion

In summary, we developed alloyed‐ligand 2D Sn‐perovskite quantum wells by incorporating a mixture of different linear oligothiophene ligands. The (4Tm)_*x*_(2T)_2−*x*_SnI_4_ alloyed‐ligand‐based materials exhibit more efficient charge transport compared to pure‐ligand materials due to the reduced interlayer distance and charge transfer energy barrier height simultaneously, further utilizing the semiconducting properties of the conjugated ligands. This improved charge transfer property allows enhancement in PV properties of devices fabricated with these conjugated thiophene ligands. More importantly, the devices display outstanding stability against thermal and moisture degradation. This work demonstrates a new strategy of fabricating organic semiconductor–perovskite hybrid materials, thus opening a new path toward the design and realization of highly stable lead‐free perovskite devices.

## Conflict of Interest

The authors declare no conflict of interest.

## Data Availability Statement

The data that support the findings of this study are available from the corresponding author upon reasonable request.

## Supporting information

Supplementary Material
