# Supplementary material for: Organic Cation Engineering for Vertical Charge Transport in Lead‐Free Perovskite Quantum Wells
Source: Small Sci. 2021 Feb 7;1(8):2000024. doi: 10.1002/smsc.202000024 (PMC11935950; doi:10.1002/smsc.202000024)
Supplement: Supplementary file 1 — Supplementary Material [file SMSC-1-2000024-s001.docx]

Copyright WILEY-VCH Verlag GmbH & Co. KGaA, 69469 Weinheim, Germany, 2018.

Supporting Information

Organic Cation Engineering for Vertical Charge Transport in Lead-Free Perovskite Quantum Wells

*Ke Ma, Sheng-Ning Hsu, Yao Gao, Zitang Wei, Linrui Jin, Blake P. Finkenauer, Libai Huang, Bryan W. Boudouris,* Jianguo Mei,* and Letian Dou**

1. **Materials and methods**

**Materials:** Tin(II) iodide (SnI_2_, 99.99%), Tin (powder, 10µm, 99%), [6,6]-Phenyl C_61_ butyric acid methyl ester (PC_61_BM, 99%), poly(triaryl amine) (PTAA), bathocuproine (BCP, 99.0%), *N*,*N*-dimethylformamide (DMF, anhydrous, 99.8%), dimethyl sulfoxide (DMSO, anhydrous, 99.9%), and diethyl ether (DEE, anhydrous, 99.8%) were purchased from Sigma-Aldrich. Poly(ethylene dioxythiophene) doped with poly(styrene sulfonate) (PEDOT:PSS) was purchased from Clevios. Gold pellets (99.999%) was purchased from Kurt J. Lesker. 4TmI and 2TI were synthesized based on our previous report^[1]^.

**Precursor solution preparation:** The (4Tm)*_x_*(2T)_2-_*_x_*SnI_4_ solution was obtained by mixing 4TmI, 2TI and SnI_2_ with molar ratio of *x*:1-*x*:1 in mixture of DMF and DMSO (*v:v* = 4:1). The concentration of SnI_2_ was kept as 0.1 M in both solutions to achieve films with 120 nm thicknesses. For thicker films, the SnI_2_ concentration was changed to 0.15 M and 0.2 M. 50% SnCl_2_ and a small amount of Sn powder was added to each precursor solution to avoid the oxidation of Sn^2+^. The mixed solutions were stirred at room temperature in a glovebox overnight. Solutions were left undisturbed for 30 min before being used in order to precipitate any Sn powder.

**2D perovskite film deposition:** To deposit (4Tm)_2_SnI_4_ and (4Tm)(2T)SnI_4_ films on either ITO substrates or PEDOT:PSS films, pre-treatment of the substrates is required. For the PEDOT:PSS substrate, 3 min of UV-ozone treatment was applied. Then the substrate was quickly moved into the glovebox, and DMF (80 µL) was spin-coated at 4000 rpm. for 10s to improve the wettability of the surface. Immediately after the DMF wetting treatment, perovskite precursor solution (25 µL) was dropped on the substrate and spin-coated at 4000 rpm. for 30s. diethyl ether (200 µL) was dropped onto the substrate around 15 s before the spinning stopped. The film was placed on a hot plate and annealed at 60 °C for 2min, then 100 °C for 10 min. For the ITO substrates, the UV-ozone treatment was extended to 15 min, while all the other process remained unchanged. The above process was used for the fabrication of devices and most of the films for characterization. The films with different thicknesses were prepared with adjusted rotation rates and precursor concentrations. The film with larger grain size was prepared through changing the annealing temperature to 180 °C.

**Device fabrication:** All ITO substrates were cleaned sequentially with a cleaning agent in deionized water, pure water, acetone, and ethanol (in a sequential manner) using ultrasonic water bath, and then dried with compressed nitrogen. The ITO substrates were first treated with UV-ozone for 30 min. The PEDOT:PSS solution was filtered with a 0.22µm filter before use. The filtered solution was spin-coated on the ITO substrate at 5,000 rpm for 40 s and annealed at 170 °C for 20 min in air. The ITO/PEDOT:PSS film was cooled to room temperature and moved into the glovebox. 2D perovskite films were fabricated with the method described above. After cooling the perovskite film to room temperature, the electron transport layer, PCBM, in a chlorobenzene solution (20mg mL^-1^) was spin-coated onto the perovskite film at 2,000 rpm for 30 s. The film was place on hot plate at 80 °C for 2 min to remove the solvent in full. Then, the hole-blocking layer, BCP (2mg mL^-1^ in isopropanol), was deposited onto the PCBM layer through spin-coating at 2,000 rpm for 30 s and dried again at 80 °C for 2 min. Finally, the device was transferred to a thermal evaporator to evaporate 60 nm Au at a pressure of 1 × 10^-6^ Pa. The FASnI_3_ films used for stability test were fabricated according to the reported method^[2]^.

**Device Characterization:** The photovoltaic output of all devices was measured under AM1.5G illumination using a solar simulator (Enlitech SS-F5-3A) calibrated using a standard Si photodiode (calibrated and certified by Enlitech). The *J-V* curves were obtained using a Keithley 2450 sourcemeter. The measurement was performed in a glovebox at room temperature by scanning the voltage from +0.8 V to –0.1 V (reverse scan) and –0.1 V to +0.8 V (forward scan) with a step of 0.02 V and a 2 ms dwell time. The device area was 0.11 cm^2^, determined by the mask. The light intensity (*ϕ*)-dependent *V_OC_* property was studied based on the relation $V_{OC}\propto\left( {k_{B}T}/q \right)\ln\varphi$, where *k_B_* is Boltzmann’s constant, *Τ *is Kelvin temperature, and $q$ is the elementary charge.

**Hole-only device fabrication, measurement, and calculation:** The hole-only devices were fabricated with a method similar to the PV device fabrication strategy. After coating the perovskite layer, a thin layer of PTAA (i.e., instead of PCBM) was coated on the perovskite. PTAA in a chlorobenzene solution (10 mg mL^-1^) was spin-coated onto the perovskite film at 6,000 rpm for 30 s, followed by drying at 80 °C for 2 min. Then 60 nm of Au was evaporated on to the device at a pressure of 1 × 10^-6^ Pa. All devices were measured from 0 to +10 V with step size of 0.02 V under dark condition using Keithley 2450 source meter unit. The hole-trap state density was calculated based on the following equation^[2,3]^.

$$N_{t}=\frac{2\varepsilon_{0}\varepsilon_{r}V_{TFL}}{qL^{2}}$$

Here, *L* is the thickness of perovskite film (i.e., 120nm), *ε_r_* (i.e., 25) is the relative dielectric constant, *ε_0_* is the vacuum permittivity, *q* is the elemental charge and *V_TFL_* is the onset voltage of the trap filled limit region.

The hole mobility values of 2D perovskite films were calculated by fitting the curve using the Mott-Gurney law in Child’s regime, which obeys the following equation^[4,5]^.

$$J=\frac{9\varepsilon_{0}\varepsilon_{r}\mu V^{2}}{8L^{3}}$$

Here, *μ* is the charge mobility, *V* is the applied voltage, and *J* is the current density.

**Perovskite film Characterization:** The thin film UV-vis absorption spectra were measured with an Agilent UV-Vis-NIR Cary-5000 spectrometer in transmission mode, where glass substrate was used as blank. The out-of-plan XRD of perovskite films was measured with a Rigaku Smart Lab using a Cu K$\alpha$ source ($\lambda$= 1.54056 Å) in Bragg Brentano mode. Thin film SEM images were obtained in secondary electron mode with a Hitachi S-4800 Field Emission SEM. Thin film AFM images were collected using a Bruker MultiMode 8 atomic force microscope in tapping mode. The bright-field optical images were recorded by a custom Olympus BX53 microscope. The PL imaging and spectra measurements were performed with SpectraPro HRS-300 spectrometer. The samples were excited with a light source (012-63000; X-CITE 120 REPL LAMP). The filter cube contains a bandpass filter (330-385 nm) for excitation, and a dichroic mirror (cutoff wavelength: 400 nm) for light splitting and a filter (Long pass 420 nm) for emission.

**References**

[1] Y. Gao, E. Shi, S. Deng, S. B. Shiring, J. M. Snaider, C. Liang, B. Yuan, R. Song, S. M. Janke, A. Liebman-Peláez, P. Yoo, M. Zeller, B. W. Boudouris, P. Liao, C. Zhu, V. Blum, Y. Yu, B. M. Savoie, L. Huang, L. Dou, *Nat. Chem.*, **2019**, *11*, 1151.

[2] J. Qiu, Y. Xia, Y. Zheng, W. Hui, H. Gu, W. Yuan, H. Yu, L. Chao, T. Niu, Y. Yang, X. Gao, Y. Chen, W. Huang, *ACS Energy Lett.*, **2019**, *4*, 1513.

[3] H. Ren, S. Yu, L. Chao, Y. Xia, Y. Sun, S. Zuo, F. Li, T. Niu, Y. Yang, H. Ju, B. Li, H. Du, X. Gao, J. Zhang, J. Wang, L. Zhang, Y. Chen, W. Huang, *Nat. Photonics*, **2020**, *14*, 154.

[4] E. A. Duijnstee, J. M. Ball, V. M. Le Corre, L. J. A. Koster, H. J. Snaith, J. Lim, *ACS Energy Lett.*, **2020**, *5*, 376.

[5] Q. Dong, Y. Fang, Y. Shao, P. Mulligan, J. Qiu, L. Cao, J. Huang, *Science*, **2015**, *347*, 967.

1. **Supplementary figures and tables**

**
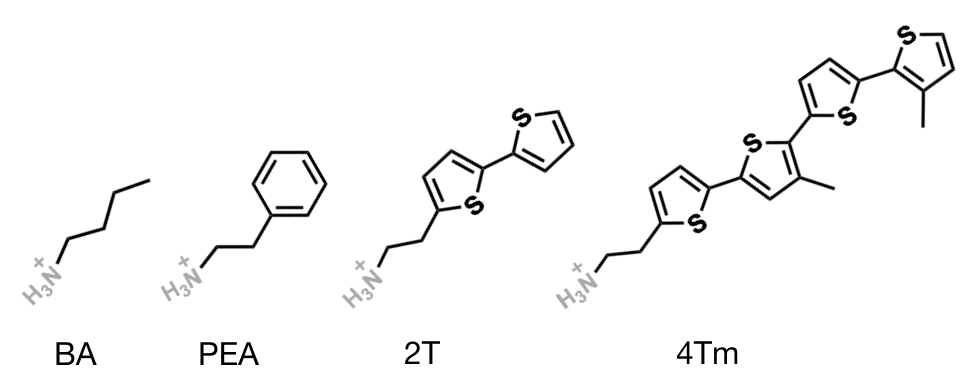
**

**Figure S1.** Chemical structures of commonly used insulating ligands BA and PEA and the semiconducting ligands used in this work: 2T and 4Tm.


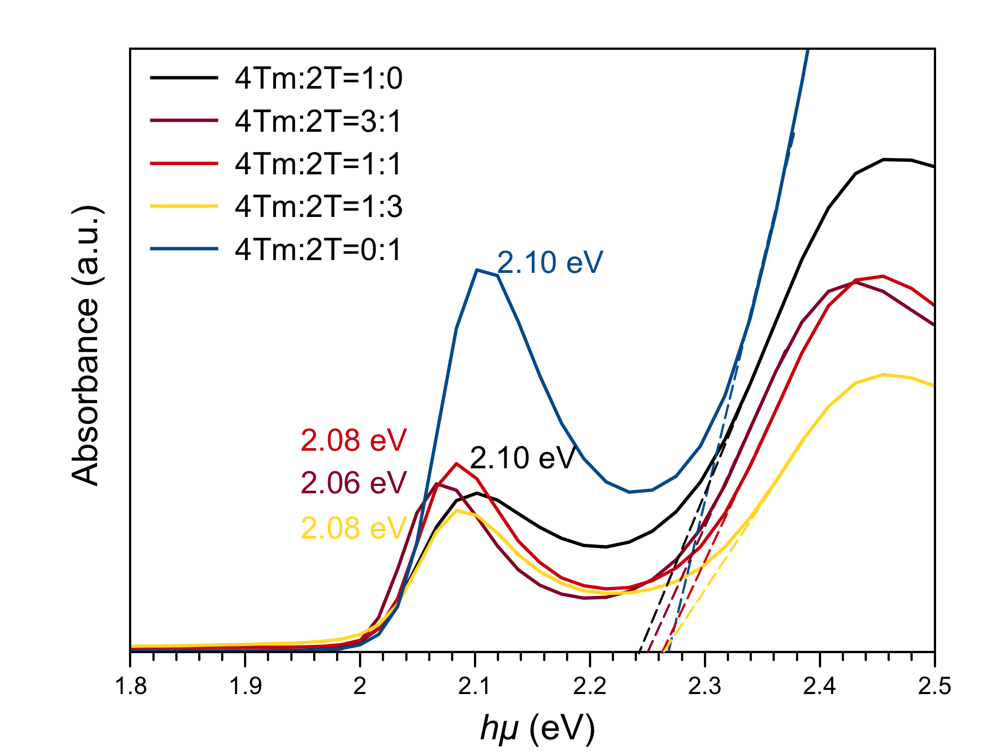


**Figure S2.** Tauc plot of (4Tm)_x_(2T)_2-x_SnI_4_ thin films, extracted from the UV-vis absorbance spectra. Exciton binding energies are extrapolated from the Tauc plot through the energy differences between the excitonic absorption peaks (the first peaks in the plot for which the energies are labeled) and the onset of continuum absorption.


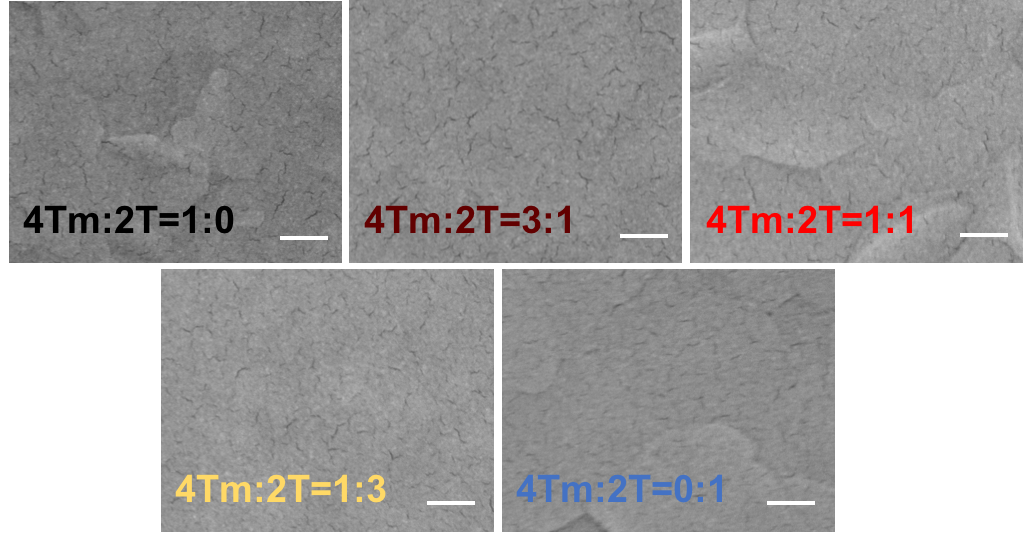


**Figure S3.** top-view SEM images of 2D perovskite thin films. Scale bar: 200nm


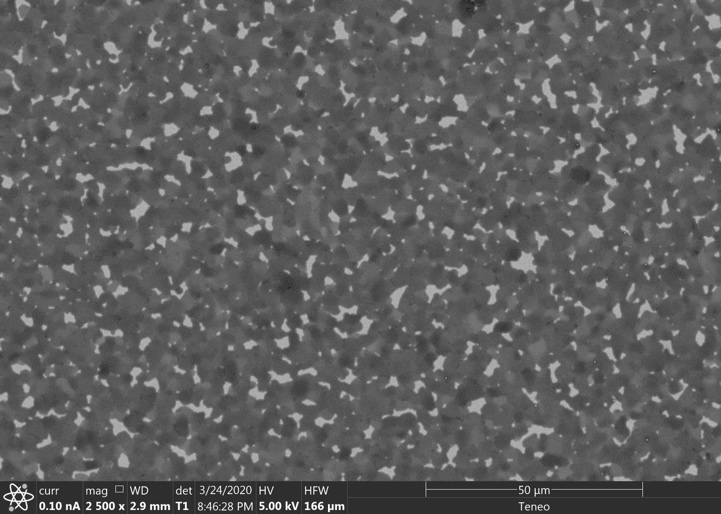


**Figure S4.** SEM image of (4Tm)_2_SnI_4_ film annealed at 180 °C. Large pinholes can be observed across the whole film. Although it obtains higher crystallinity and higher PL intensity, it is not suited for PSCs due to the potential current leakage caused by the pinholes.

**
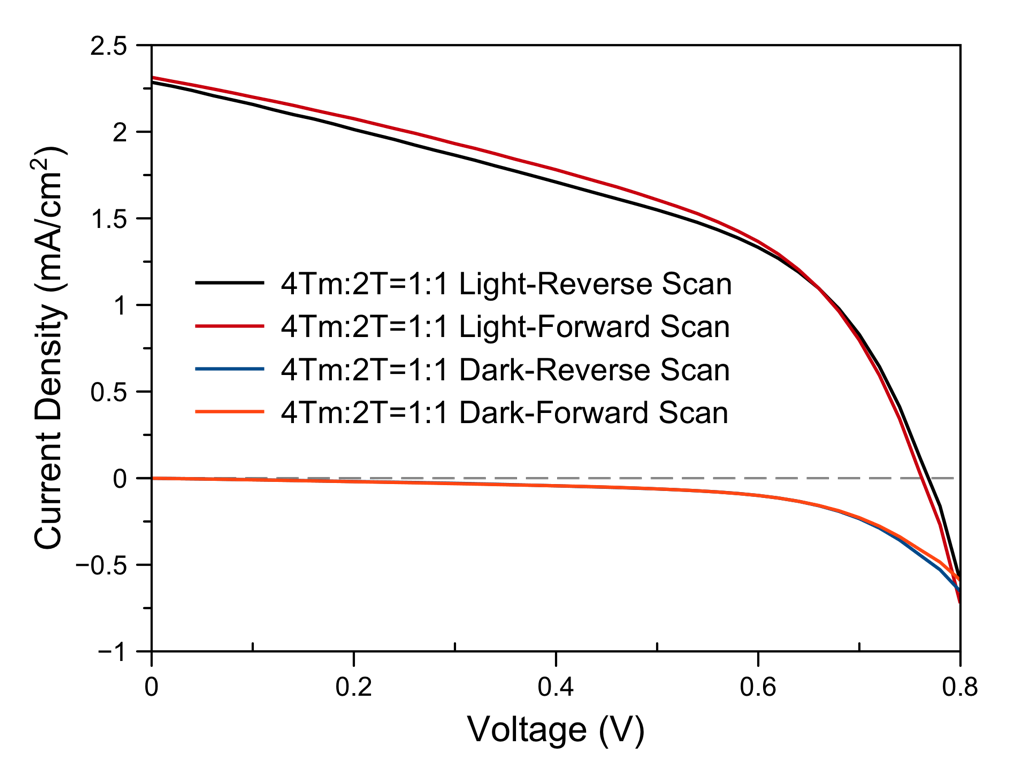
**

**Figure S5.** *J-V* curve of 1:1 device with reverse and forward scan, both under light and in the dark.


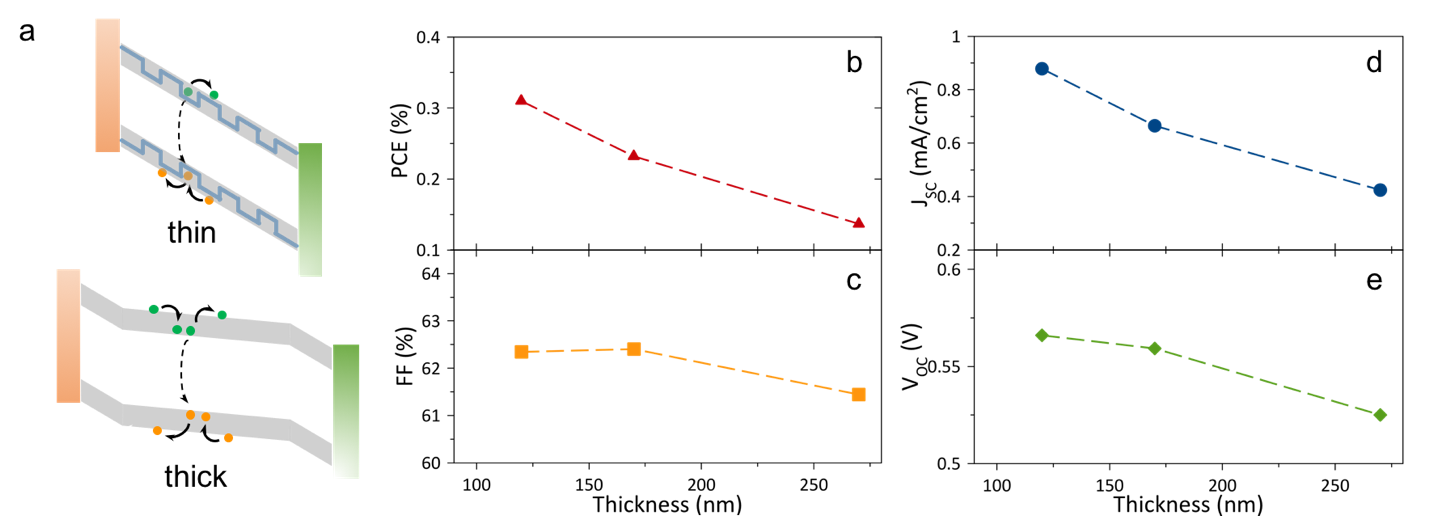


**Figure S6.** Thickness dependent charge transportation process.(a) A schematic illustration of quantum well tunneling process under different film thickness. (b) PCE, (c) *FF*, (d) *J_SC_* and (e) *V_OC_* of (4Tm)_2_SnI_4_ PSCs as a function of 2D perovskite film thickness.


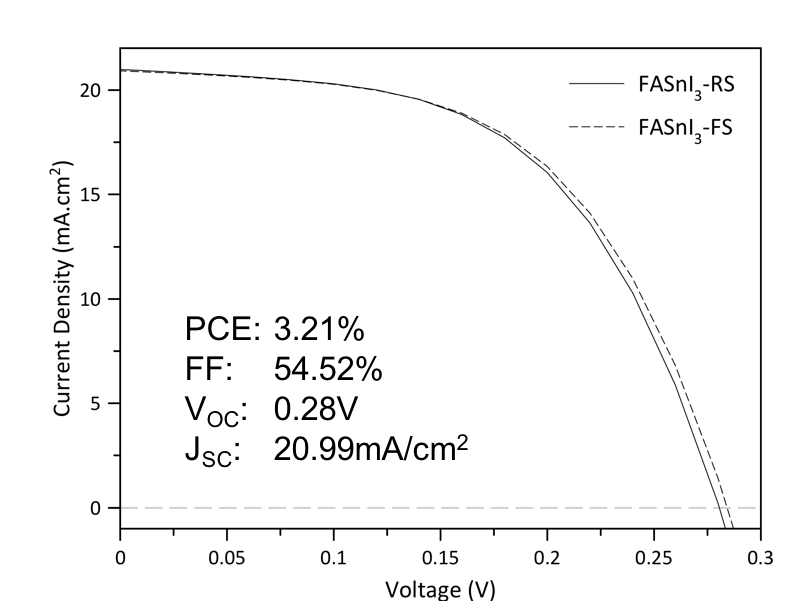


**Figure S7.** *J-V* curve of 3D FASnI_3_ device before the stability test.


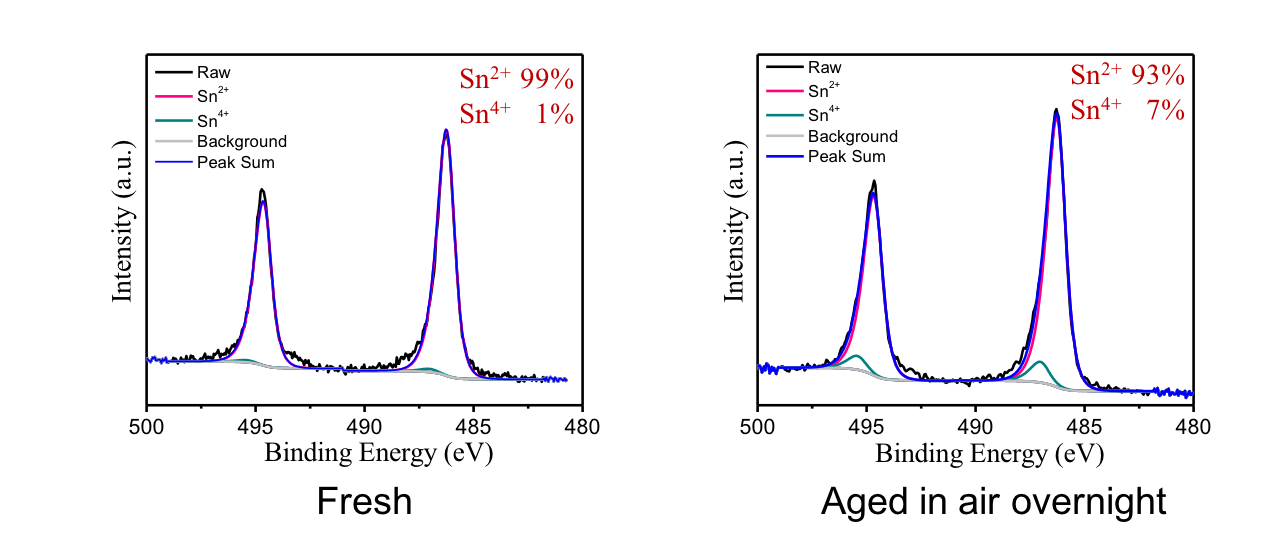


**Figure S8**. XPS spectra of (4Tm)_2_SnI_4_ film before and after aging in air for overnight duration. As shown in the spectra, the fresh film contains 99% of Sn^2+^ with 1% of oxidized form of Sn^4+^. After aging in air overnight, only 6% of Sn^2+^ is oxidized to Sn^4+^.
